# Nitric Oxide-Induced Calcium Release: Activation of Type 1 Ryanodine Receptor, a Calcium Release Channel, through Non-Enzymatic Post-Translational Modification by Nitric Oxide

**DOI:** 10.3389/fendo.2013.00142

**Published:** 2013-10-11

**Authors:** Sho Kakizawa

**Affiliations:** ^1^Department of Biological Chemistry, Graduate School of Pharmaceutical Sciences, Kyoto University, Kyoto, Japan

**Keywords:** gaseous messenger, post-translational modification, nitric oxide, ryanodine receptor, S-nitrosylation, calcium release, synaptic plasticity, Purkinje cell

## Abstract

Nitric oxide (NO) is a typical gaseous messenger involved in a wide range of biological processes. In our classical knowledge, effects of NO are largely achieved by activation of soluble guanylyl cyclase to form cyclic guanosine-3′, 5′-monophosphate. However, emerging evidences have suggested another signaling mechanism mediated by NO: “S-nitrosylation” of target proteins. S-nitrosylation is a covalent addition of an NO group to a cysteine thiol/sulfhydryl (RSH), and categorized into non-enzymatic post-translational modification (PTM) of proteins, contrasted to enzymatic PTM of proteins, such as phosphorylation mediated by various protein kinases. Very recently, we found novel intracellular calcium (Ca^2+^) mobilizing mechanism, NO-induced Ca^2+^ release (NICR) in cerebellar Purkinje cells. NICR is mediated by type 1 ryanodine receptor (RyR1), a Ca^2+^ release channel expressed in endoplasmic-reticular membrane. Furthermore, NICR is indicated to be dependent on S-nitrosylation of RyR1, and involved in synaptic plasticity in the cerebellum. In this review, molecular mechanisms and functional significance of NICR, as well as non-enzymatic PTM of proteins by gaseous signals, are described.

## Introduction

Primary structure of proteins obtained from genome analysis is not sufficient to explain their various biological functions: while it is estimated that the human genome, for example, comprises ∼27,000 genes, the total number of proteins in the human proteome is estimated at over one million. In addition to changes at the transcriptional and mRNA levels, it is now increasingly recognized that “post-translational modification (PTM) of proteins” provide important roles in a wide range of signaling pathways, including intercellular signaling pathways such as endocrine systems as well as intracellular pathways. PTMs are covalent processing events that change the properties of a protein by proteolytic cleavage or by addition of a modifying group to one or more amino acids. PTMs of proteins are indicated to be involved in various biological events through changes in protein activity, their cellular locations and dynamic interactions with other proteins (1, 2).

More than 300 different types of PTMs are currently known, and new ones are regularly discovered (3). In general, PTMs are categorized into two groups: enzymatic modification and non-enzymatic modification. Enzymatic PTM, including phosphorylation, acetylation, glycosylation, and lipidation, are demonstrated to be involved in a wide range of physiological and pathophysiological events in eukaryotic cellular systems. On the other hand, accumulation of products derived from non-enzymatic PTM is seen in various tissues of metabolic and age-related diseases such as Alzheimer’s disease, Parkinson’s disease, cataractogenesis, atherosclerosis, diabetic secondary complications, etc. (4–7). Consequently, it is thought that these accumulations are possibly causative to age-related pathology, and non-enzymatically modified proteins are considered to be useful biomarkers for these diseases (8, 9).

However, recent studies indicate that non-enzymatic PTM of proteins is also associated with physiological events. For example, S-nitrosylation by nitric oxide (NO) is now well established as a major source of NO bioactivity (10–12), and proteins shown to be modified *in situ* by S-nitrosylation (SNO-proteins) participate in a wide range of biological process including those involved in cellular trafficking (13), muscle contractility (14), apoptosis (15, 16), and circulation (17). In addition to non-enzymatic PTMs by reducing molecules, such as glycation by glucose, non-enzymatic modification by gaseous messengers is now attracting much attention.

## Post-Translational Modification by Gaseous Messengers

A gas is a state of matter different from either the liquid or solid states. Gases possess the ability to diffuse readily in different materials and distribute uniformly within a defined space. Biological gases are assumed to diffuse freely across biologic membranes (18). Thus, gases do not bind to cell surface receptors, and do not require the intermediation of conventional membrane receptors and second messenger machinery such as G-proteins and adenylyl cyclase (19). Instead, the gases directly interact with targets, such as guanylyl cyclase (20). In addition to the reactions with metal centers of metalloproteins (e.g., hemoglobin), significant proportion of the direct action of gaseous messengers is mediated through non-enzymatic PTM of proteins, such as S-nitrosylation by NO and sulfhydration of hydrogen sulfide (H_2_S) (21–23).

Probably most prevalent is the “S-nitrosylation” by NO. NO is produced enzymatically in cells expressing NO synthase (NOS) (19). Addition of NO group to the thiol side chain of cysteine residues within proteins and peptides is termed S-nitrosylation. Furthermore, peroxynitrite, produced by the reaction of NO with superoxide, is demonstrated to regulate cellular signaling (24). Peroxynitrite reacts with several amino acids. Cysteine, methionine, and tryptophan react directly, whereas tyrosine, phenylalanine, and histidine are modified through intermediary secondary species (25). Therefore, emerging evidence have indicated that non-enzymatic PTM of proteins by gaseous messengers is involved in physiological and pathological events in various biological systems (26).

## Functional Modification of Ryanodine Receptors by S-Nitrosylation

Skeletal and cardiac muscles mainly express neuronal NOS (nNOS) and endothelial NOS (eNOS), respectively (27, 28), and endogenously produced NO can promote two physiological functions of these muscles. The first is to induce relaxation through the cGMP signaling pathway (29, 30). The second is to modulate increases in contraction that are dependent on reactive oxygen intermediates and independent on cGMP (27). Stoyanovsky et al. (31) thus examined effects of NO-related compounds on Ca^2+^ release from sarcoplasmic reticulum (SR) isolated from skeletal and cardiac muscles (31). The compounds, such as *S*-nitrosocysteine (cysNO), *S*-nitroso-*N*-acetylpenicillamine (SNAP), and S-nitrosylated glutathione (GSNO), induced Ca^2+^ release from the isolated SR vesicles. Correspondingly, application of SNAP increased open probability of SR channels in lipid bilayer (31). The effects of NO-related compounds on the activity of Ca^2+^ release channels were observed in subsequent study: Xu et al. (14) showed that GSNO and cysNO increased open probability of cardiac Ca^2+^ release channel in lipid bilayer (14). In this study, the rise in open probability of cardiac Ca^2+^ release channel was accompanied with the increased amount of *S*-nitrosothiol group per channel protein, the result suggesting the activation of cardiac Ca^2+^ release channels by S-nitrosylation (14). However, as is demonstrated later, NO or 1-hydroxy-2-oxo-3-(*N*-ethyl-2-aminoethyl)-3-ethyl-1-triazene (NOC12) (an NO donor) do not activate or *S*-nitrosylate RyR2 while GSNO induce activation and S-nitrosylation of RyR2 (32). Thus, at that time, cardiac Ca^2+^ release channels, possibly RyR2, was suggested to be activated by GSNO-induced S-nitrosylation of the channel (14).

Subsequently, NO-induced S-nitrosylation of skeletal Ca^2+^ release channel was demonstrated. In these studies, NO-sensitivity of the channels had been studied extensively in ambient O_2_ tension (pO_2_ ∼150 mmHg), whereas tissue pO_2_ is ∼10–20 mmHg and even lower in exercising muscle (33, 34). Eu et al. (35) showed that Ca^2+^ release channel in SR isolated from skeletal muscle, possibly type 1 ryanodine receptor (RyR1), was activated and S-nitrosylated by submicromolar concentration of NO (35). This modification of the channel was induced restrictedly in the case that pO_2_ was at tissue level (∼10 mmHg) but not at ambient level (∼150 mmHg). However, when the concentration of NO was increased to micromolar ranges, the channels were activated by NO even in the ambient O_2_ levels (35). Moreover, unlike the case in NO, activation of the channels by NOC12 or GSNO was indicated to be insensitive to pO_2_ levels: NOC12 and GSNO activated skeletal Ca^2+^ channels in SR at ambient O_2_ levels even when the concentration of these compounds were low enough and the estimated levels of NO produced from these compounds were submicromolar ranges (36).

Eu et al. (35) also estimated that only one cysteine in RyR1 was S-nitrosylated by submicromolar concentrations of NO at tissue O_2_ level (35). Subsequently, the target of S-nitrosylation was identified as Cys3635: single-cite C3635A-mutaion in RyR1 abolished NO-induced S-nitrosylation of the mutated channels expressed in HEK 293 cells (37). In addition, the NO-induced rise in open probability was abolished in the C3635A-mutant channels in lipid bilayer (36). C3635 is intercalated within the hydrophobic calmodulin (CaM)-binding domain of RyR1, and thereby S-nitrosylation of C3635 is thought to reverse channel inhibition by CaM (38–40). Furthermore, C3635 was indicated to be not required for the activation of the channel by GSNO (36). Taken together, these observations suggest that NO, NOC12, and GSNO activate the redox-sensitive RyR1 channel by different mechanisms, and the effect of O_2_ tension on S-nitrosylation by NO is best rationalized by an allosteric mechanism (36).

It is also demonstrated that RyR2 is not activated by NO and the response of RyR3 to NO is much smaller than that of RyR1 (32, 41). Because the important cysteine (i.e., C3635 of rabbit RyR1) is conserved among all RyR subtypes, the three-dimensional structure around the critical cysteine residue may be important for the subtype specificity of S-nitrosylation or channel gating. However, this mechanism requires further clarification.

## Biological Function of RyR1

Type 1 ryanodine receptor is an intracellular calcium release channel involved in regulation of cytosolic calcium levels. The highest levels of RyR1 expression are observed in skeletal muscle, and significantly higher levels of RyR1 mRNA are seen in the esophagus and testis, when compared to other tissues (42). In addition, lower amount are found in the spleen, gut kidney, stomach, submaxillary gland, thymus, adrenal gland, and ovary. In mammalian brain, RyR1 mRNA is prominent in the cerebellar Purkinje cell (PC) layer and dentate dyrus in the hippocampal region (43). It is well known that RyR1 is essential for contraction of skeletal muscles. However, the function of RyR1 has yet to be clarified in the other tissues, because the mutant deficient in RyR1 gene shows postnatal lethality (44). Furthermore, RyR1 is demonstrated to be physiologically regulated by protein–protein interaction to voltage-gated calcium channels in skeletal muscle cells (45). Although RyR1 is also expressed in the brain, as is described above, such tight Ca^2+^ channel-mediated regulation of intracellular Ca^2+^ release through RyR1, as seen in skeletal muscle cells, is absent in central neurons (46). Therefore, further study into the regulatory mechanisms and functions of RyR1 in the brain is warranted.

## NO-Induced Ca^2+^ Release Induced by Neuronal Activity

As is described so far, the redox regulation of RyRs were extensively studied using *in vitro* experimental systems, especially in lipid bilayer and SR isolated from skeletal and cardiac muscles. On the other hand, involvement of S-nitrosylation of RyR in Ca^2+^ release in living cells and physiological function of the channel modulation by endogenous NO have yet to be demonstrated, although increased open probability of RyR1 by S-nitrosylation was suggested to enhance Ca^2+^ leakage from skeletal muscle Ca^2+^ stores (SR) under pathological conditions (47, 48).

Involvement of S-nitrosylation in neuronal function has been suggested in the cerebellar cortex. PCs, the principal and solely output neurons in the cerebellar cortex, receive two types of excitatory (glutamatergic) inputs: climbing fiber, originate from inferior olive, and parallel fiber (PF), axon of cerebellar granule cells (49). The PF-to-PC synapse (PF synapse) is extensively studied, because many studies indicate that long-term depression (LTD), a kind of synaptic plasticity, of PF synapse is a cellular basis for the cerebellar-dependent learning such as eyeblink conditioning (49–51). In addition to LTD, long-term potentiation (LTP) is observed in PF synapse (PF-LTP). When PF are repeatedly stimulated, the current response of PF synapse is potentiated for 30 min, at least. Three groups demonstrated that the PF-LTP is dependent on NO signals (52–54). Furthermore, although the protocol (the stimulus pattern) for the LTP induction are slightly different between Lev-Ram et al. (53) and Namiki et al. (54), both groups demonstrated that this NO-dependent LTP is insensitive to 1H-[1,2,4]oxadiazolo[4,3-a]quinoxalin-1-one (ODQ), a selective inhibitor of sGC activation by NO (53, 54). Thus, PF-LTP is indicated to be dependent on signaling pathways mediated by S-nitrosylation. Furthermore, in their preliminary experiments, Kakizawa et al. observed that PF-LTP was dependent also on intracellular Ca^2+^ signals. Taken together, PF-LTP was indicated to be dependent on S-nitrosylation and Ca^2+^ signals, and these results lead to our hypothesis: S-nitrosylation-mediated Ca^2+^ release is induced by PF activity and involved in the induction of PF-LTP.

**Figure 1:**
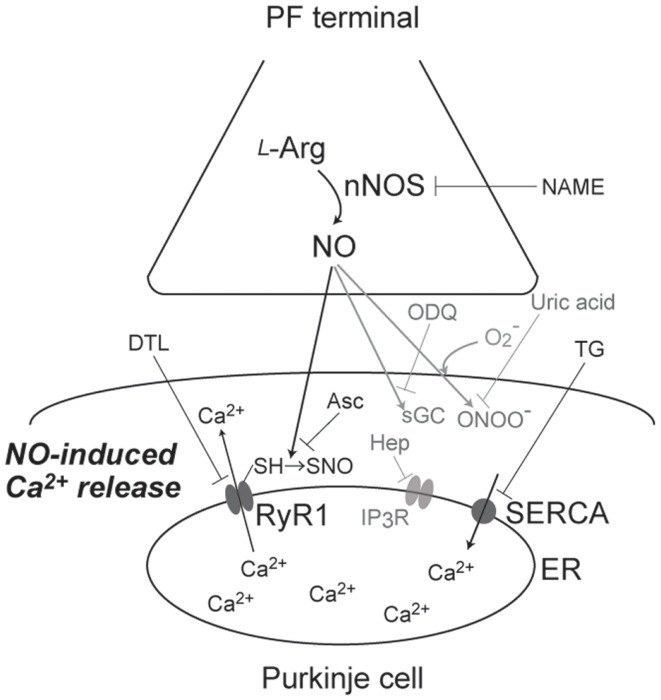
**A schematic diagram of signaling pathways for NO-induced Ca^2+^ release**. NO, produced at PF terminal, diffuse and induce Ca^2+^ increase in Purkinje cell. This NO-induced Ca^2+^ increase is sensitive to NAME (broad NOS inhibitor), Asc (reducing reagent), DTL (RyR1 antagonist), and TG (SERCA inhibitor), but not affected by ODQ (sGC inhibitor), heparin (IP_3_R antagonist), and uric acid (peroxynitrite scavenger). Thus, the Ca^2+^ increase is revealed to be Ca^2+^ release mediated by S-nitrosylation of RyR1 by endogenous NO.

First, Kakizawa et al. (41) demonstrated that bath application of 1-hydroxy-2-oxo-3-(*N*-methyl-3-aminopropyl)-3-methyl-1-triazene (NOC7), an NO donor, induced Ca^2+^ elevation in cerebellar PC in acute slice preparation from young-adult (1- to 2-month-old) mice (41). Subsequently, this NO-induced Ca^2+^ elevation was revealed to be Ca^2+^ release mediated by RyR1: the NO-induced Ca^2+^ increase was abolished by thapsigargin, cyclopiazonic acid (CPA) [inhibitors of sarco/endoplasmic-reticulum Ca^2+^ ATPase (SERCA)], and dantrolene (a specific inhibitor for RyR1), but insensitive to chelating the extracellular Ca^2+^ and heparin (a specific inhibitor for IP_3_Rs) (Figure 1). Involvement of RyR1 was further confirmed by impaired NO-induced Ca^2+^ elevation in PCs in RyR1-knockout mice (44). Moreover, RyR1 was indicated to be necessary and sufficient for NO-induced Ca^2+^ increase by the experiment using HEK 293 cells, expressing little endogenous RyRs: NO-induced Ca^2+^ elevation was observed only in the cells expressing exogenous RyR1, identified by Ca^2+^ response to caffeine, a well-known agonist of RyRs. Furthermore, NO-induced Ca^2+^ increase was indicated to be insensitive to ODQ, a sGC inhibitor. The result suggested that the Ca^2+^ increase is independent on sGC-mediated pathways, including activation of RyRs by cyclic ADP ribose, whose formation is induced by cGMP (55). Instead, the result indirectly suggested that NO-induced Ca^2+^ increase is dependent on S-nitrosylation of proteins (Figure 1). Correspondingly, biochemical analysis indicated that NO-induced Ca^2+^ elevation was accompanied with the transient increase in S-nitrosylation levels of endogenous RyR1 in cerebellar slices. NO-induced increase in S-nitrosylation level was also observed in exogenous RyR1 expressed in HEK cells. Thus, NO-induced Ca^2+^ elevation was revealed to be Ca^2+^ release mediated by RyR1 (Figure 1), and was named “NO-induced Ca^2+^ release (NICR)” (41).

Subsequently, Kakizawa et al. (41) examined induction of NICR by physiological patterns of neuronal activity. When the burst stimulus (BS) inducing PF-LTP was applied to PF, Ca^2+^ levels in PCs were transiently but clearly elevated. This BS-induced Ca^2+^ increase was indicated to be Ca^2+^ release mediated by RyR1, because the Ca^2+^ increase was abolished by bath application of thapsigargin, CPA, and dantrolene. Furthermore, BS-induced Ca^2+^ elevation was inhibited by NG-nitro-l-arginine methyl ester (l-NAME), a broad antagonist of NOSs, and abolished also in nNOS-knockout mice (56). Taken together, BS-induced Ca^2+^ elevation was revealed to be dependent on both NO signals and Ca^2+^ release through RyR1 (Figure 1). Thus, BS-induced Ca^2+^ increase is indicated to be NICR, and NICR is demonstrated to be induced by physiological patterns of neuronal activity.

Neuronal activity in conjunction with certain forms of synaptic plasticity may be associated with superoxide generation, and NO generation leads to peroxynitrite formation when superoxide is simultaneously generated (57, 58). Because peroxynitrite is known to regulate cell signaling via molecular modifications, including protein nitration (57, 58), it seems possible that NICR is affected by peroxynitrite produced from endogenous NO in the presence of superoxide. Therefore, Kakizawa et al. (59) examined the potential role of peroxynitrite in the BS-induced Ca^2+^ release and LTP (59). Bath application of the peroxynitrite scavenger, uric acid, and the peroxynitrite decomposition catalyst, 5,10,15,20-tetrakis(4-sulfonatophenyl)porphyrinato iron(III) (FeTPPS), had no effect on BS-induced Ca^2+^ increase), although the concentration of uric acid (100 μM) and FeTPPS (10 μM) were thought to be high enough. In accordance with the insensitivity of NICR to these reagents, neither uric acid nor FeTPPs impaired the induction of PF-LTP induced by BS. These results do not support the involvement of peroxynitrite in NICR and PF-LTP induction, and NICR is indicated to by induced directly by endogenous NO (Figure 1).

## Physiological Function of NICR

Because the BS, which induces NICR, has been already indicated to induce PF-LTP (54), it is strongly suggested that NICR is involved in the induction of PF-LTP. Actually, all manipulations that inhibit NICR (application of l-NAME, thapsigargin, and dantrolene; see also Figure 1) abolished PF-LTP in PCs. In addition, neither PF-LTP nor NICR was induced in the cerebellum of nNOS-knockout mice. Furthermore, both PF-LTP and NICR were inhibited by intracellular application of ascorbic acid from patch pipette (Figure 1). Because ascorbic acid is a reducing agent, the results also support the idea that S-nitrosylation is required for the induction of NICR as well as PF-LTP. On the other hand, bath application of uric acid, a scavenger of peroxynitrite (ONOO^−^, produced by reaction of NO with superoxide), or pipette application of heparin inhibited neither NICR nor PF-LTP. Taken together, NICR is revealed to be induced by physiological activity of PF and essential for the induction of PF-LTP, and these observations strongly indicate that Ca^2+^ release mediated by S-nitrosylation of RyR1 is induced in living cells and has physiological function(s) (41) (Figure 1).

## Perspectives: Possible Induction and Physiological Function of NICR *in vivo*

In the study by Kakizawa et al. (41), NICR was induced by physiological patterns of neuronal activity (BS to PF) in artificial cerebrospinal fluid (ACSF) bubbled with 95% O_2_/5% CO_2_. In the component of PF-PC synapse, expression of nNOS is observed in PFs and NO is thought to be released from PF terminal (60, 61). Thus, NO, produced at PF in response to the neuronal activity, is thought to diffuse and induce S-nitrosylation of RyR1 in PCs. As is already demonstrated by Eu et al. (35), RyR1 is S-nitrosylated in the ambient pO_2_ levels only when the concentration of NO is micromolar level (35). Is the level of NO produced by the BS inducing NICR and PF-LTP micromolar range? Using the NO-sensitive fluorescent probe, Namiki et al. (54) estimated the BS-induced NO level in PCs in the cerebellar slice (54). The NO concentration induced by the BS was estimated to the order of ∼5 μM. Correspondingly, in the cerebellar PCs, NICR was induced when NOC7 was higher than 10 μM (Kakizawa et al., unpublished data), and 10 μM NOC7 is estimated to yield ∼1 μM NO (54). Thus, NICR is suggested to be induced by physiological patterns of neuronal activity, which induces PF-LTP, through the micromolar levels of NO in the ambient pO_2_ condition. On the other hand, it is still possible that the level of NO required for the induction of NICR and PF-LTP is overestimated. because pO_2_ at tissue level (∼10 mmHg) is much lower than the ambient level (∼150 mmHg), and the submicromolar levels of NO is indicated to induce S-nitrosylation and activation of RyR1 at the tissue pO_2_ levels (35).

Expressions of RyR1 as well as NOS (especially nNOS) are observed in various regions of the brain (43, 60). Furthermore, expression of RyR1 is demonstrated in various tissues other than brain and skeletal muscles, such as digestive tissues and reproductive tissues (42). Therefore, NICR may have a wide range of physiological functions in these tissues.

## Conflict of Interest Statement

The author declares that the research was conducted in the absence of any commercial or financial relationships that could be construed as a potential conflict of interest.
